# Climate data sonification and visualization: An analysis of topics, aesthetics, and characteristics in 32 recent projects

**DOI:** 10.3389/fpsyg.2022.1020102

**Published:** 2023-01-25

**Authors:** PerMagnus Lindborg, Sara Lenzi, Manni Chen

**Affiliations:** ^1^SoundLab, School of Creative Media, City University of Hong Kong, Kowloon, Hong Kong SAR, China; ^2^Critical Alarms Laboratory, Faculty of Industrial Design Engineering, Delft University of Technology, Delft, Netherlands

**Keywords:** climate data, science communication, sonification, visualization, aesthetic perspective, circumplexity, exploratory factor analysis, lexical diversity

## Abstract

**Introduction:**

It has proven a hard challenge to stimulate climate action with climate data. While scientists communicate through words, numbers, and diagrams, artists use movement, images, and sound. Sonification, the translation of data into sound, and visualization, offer techniques for representing climate data with often innovative and exciting results. The concept of sonification was initially defined in terms of engineering, and while this view remains dominant, researchers increasingly make use of knowledge from electroacoustic music (EAM) to make sonifications more convincing.

**Methods:**

The Aesthetic Perspective Space (APS) is a two-dimensional model that bridges utilitarian-oriented sonification and music. We started with a review of 395 sonification projects, from which a corpus of 32 that target climate change was chosen; a subset of 18 also integrate visualization of the data. To clarify relationships with climate data sources, we determined topics and subtopics in a hierarchical classification. Media duration and lexical diversity in descriptions were determined. We developed a protocol to span the APS dimensions, Intentionality and Indexicality, and evaluated its circumplexity.

**Results:**

We constructed 25 scales to cover a range of qualitative characteristics applicable to sonification and sonification-visualization projects, and through exploratory factor analysis, identified five essential aspects of the project descriptions, labeled Action, Technical, Context, Perspective, and Visualization. Through linear regression modeling, we investigated the prediction of aesthetic perspective from essential aspects, media duration, and lexical diversity. Significant regressions across the corpus were identified for Perspective (ß = 0.41^***^) and lexical diversity (ß = −0.23^*^) on Intentionality, and for Perspective (ß = 0.36^***^) and Duration (logarithmic; ß = −0.25^*^) on Indexicality.

**Discussion:**

We discuss how these relationships play out in specific projects, also within the corpus subset that integrated data visualization, as well as broader implications of aesthetics on design techniques for multimodal representations aimed at conveying scientific data. Our approach is informed by the ongoing discussion in sound design and auditory perception research communities on the relationship between sonification and EAM. Through its analysis of topics, qualitative characteristics, and aesthetics across a range of projects, our study contributes to the development of empirically founded design techniques, applicable to climate science communication and other fields.

## 1. Introduction

Increasingly, researchers are asked to be more than knowledge-creators within their field of expertise, and “envisage the optimal processes and techniques for translating data into understandable, consumable modes of representation for audiences to digest” (Chandler et al., 2015). It has proven a hard challenge to present climate science to convince not only decision-makers but also the general public. Another challenge is the “information deficit” fallacy, which arises when abundant information is assumed to lead to better understanding simply by existing (e.g., in scientific publications) but fails in its purpose because the meaning is “inaccessible and misaligned with the needs of different audiences” (Jacobs et al., 2017). This is a conundrum that plays into the hands of climate denialists. While scientists mainly communicate through words, numbers, and diagrams, artists and designers may use movement, images, sound, and sculpture. Artistically inclined researchers have proposed to “move away from…static visualizations, and visual narratives with simplistic messages” (Jacobs et al., 2017) and instead communicate through “data displays that embed,” and embody, knowledge about climate science “in more sensory, tangible and visceral representations” which will make the scientific data “come alive” (Polli, 2011). This is possible by creating “dynamic and performative experiences of scientific data…that support engagement with issues of complexity, uncertainty and risk” (Jacobs et al., 2017). Interdisciplinary projects are often based on the notion that both art and science are “founded essentially on curiosity, but the challenge and the difficulty reside in the reality of bringing together contrasting methodologies that frequently use very different written and visual languages” (Ruddock et al., 2012).

Sonification, the translation of data into sound, and visualization, the translation of data into light, offer techniques for designing sonic and visual representations of scientific data in ways that can often be highly innovative and exciting. Data visualization as a discipline has been systematized over the past century with the definition of standardized methods for the translation of data into visuals such as static images, animated images, and, more recently interactive web applications (Bertin, 1967; Munzner, 2014). Meanwhile, sonification is still an emerging discipline that struggles to define its boundaries, its impacts and more importantly, shared methods, processes, and tools for the mapping of data to sound (Lenzi, 2021). The relationship between standardized data visualization strategies such as Bértin's “visual variables” and sonification strategies is a growing Research Topic within the sonification community (Enge et al., 2021; Caiola et al., 2022).

It is a major design challenge to create sonifications that are “not only effective at communicating information but which are sufficiently engaging to engender sustained attention. Sonification may be ineffective if the rendered sound appears arbitrary to the listener in relation to the underlying data. The design task then becomes about finding a suitable fit between communicational efficacy and appropriate aesthetic character” (Vickers et al., 2017, p. 2). When sonification emerged as a field of study around three decades ago (Kramer, 1994; Hermann et al., 2011), it was defined in terms of engineering and utilitarian purposes. Since then, researchers in the field of auditory display have argued, sometimes vividly, whether to make use of design principles developed for sound art and electroacoustic music composition (Barrass and Vickers, 2011), placing a stronger focus on aesthetics and systematic evaluation (Bonet, 2021), or stay true to Kramer's original concept. The first author of the present study has proposed to extend the definition of sonification as “any technique that translates data into non-speech sound, with a systematic, describable, and reproducible method, in order to reveal or facilitate communication, interpretation, or discovery of meaning that is latent in the data, having a practical, artistic, or scientific purpose” (Liew and Lindborg, 2020). A parallel definition was proposed for visualization (see also Lankow et al., 2012, p. 20). However, note that while sonification defined this way embraces art, it still excludes speech [as Kramer (1994) did in his seminal paper]. Speech contains acoustic symbols (utterances) that convey semantic meaning within a given context (language). In contrast, it is hard to uphold that “visualization” should exclude visual symbols such as text, numbers, emojis etc., at least in practice. The exclusion of “speech sound” in sonification has been questioned (Boehringer, 2022, in review).

Recent research shows that sonification can overcome barriers in science communication, because “the translation from data into audio reveals changing variables to the listener through changes in sonic dimensions, such as frequency, pitch, amplitude, and location in the stereo field. In musical contexts, data can map to these sonic dimensions, as well as higher-order musical dimensions, such as tempo, form, and timbre” (Sawe et al., 2020). A recent review study on sonification strategies in astronomical research (Zanella et al., 2022) highlights the added value of using sound to increase the accessibility of scientific knowledge, especially for the engagement of a visually impaired non-expert audience. In a meta-study, Dubus and Bresin (2013) charted strategies for the sonification of real-world physical processes. The central design strategy is referred to as “parameter mapping.” The parameters in question describe the input and output spaces; the input might be time stamped and geo-tagged measurements of the environment such as ocean temperature, atmospheric CO_2_ levels, polar ice coverage, biodiversity loss, and so on, and the output are the parameters of sound generation (Walker and Nees, 2011), connected by a “mapping” network that describes the relationships between the two. This perspective, however, does not speak about qualitative characteristics of either input or output, or of the influence of sometimes nebulous information accompanying the sonification itself: like the remora fish that accompany large sharks. While the actual media itself is certainly the focus of our attention (in the present study: audio recordings of sonifications, or movie recordings of concurrent sonification-visualizations), the output as a whole can be a very complex entity. In many cases it is a sprawling set of informative documents, technical descriptions, photography, design drawings, iterative process output, contextual descriptions, published articles, and more. Moreover, there is no standardized way of documenting such projects, and each new project will define the form, shape, and size of presentation materials necessary to bring its communicative purposes across to audiences. How can we evaluate such complexity across several projects?

In the present study, the question of how to understand the relationship between aesthetic perspective and qualitative characteristics of projects is central. Aesthetic judgement is considered to be a perceptual-cognitive mechanism of higher order (consider the BRECVEMA framework, Juslin, 2013). Kramer (1994) was keenly aware that certain perceptual qualities are essential for a data sonification to be received and understood as meaningful. He argued for scientific evaluation criteria—systematicity, objectivity, and replicability—so that sonification might “be used as a scientific method” (Hermann, 2008). This resulted in sonification, as a discipline or technique, came to be closely associated with information engineering. In reaction, other researchers argued that data sonification could benefit from knowledge about auditory perception gained in artistic fields, notably sound art and electroacoustic composition (Vickers and Hogg, 2006; Barrass and Vickers, 2011; Vickers et al., 2017). Research in aesthetic appreciation in the arts has a very long history. The attention to everyday experiences and environments is more recent (Berlyne, 1974 set the tone), as is the systematic study of aesthetic emotions (Schindler et al., 2017; Menninghaus et al., 2019), the measurement of qualia such beauty, awe, and interestingness (Silvia, 2005; Ramakrishnan and Greenwood, 2009), and in particular, aesthetics within a framework of music emotions (Juslin, 2019). As a tool to chart the perceptual relationships between sonification and other sonic design techniques, Vickers and Hogg (2006) proposed the Aesthetic Perspective Space (APS; see also Vickers et al., 2017). It is a theoretical construct, a two-dimensional circumplex model, aimed at bridging auditory display and electroacoustic music. APS presents itself as a circumplex with two axes, labeled Intentionality and Indexicality. The first dimension describes purpose, and is anchored by the concepts of Ars Informatica (utilitarian) and Ars Musica (artistic). The second dimension describes the nature of sonic materials, whether the sonic material points toward Concrete or Abstract ontologies. Intentionality is a gradient of the designer's intention in taking “deliberate decisions to address specific needs, in a given context and with a purpose, when transforming data into sound” (Lenzi and Ciuccarelli, 2020), while Indexicality indicates the facility of causal inference: how much a sound “sounds like the thing that made it” (Vickers and Hogg, 2006, p. 213). Section 4.1.3 below discusses APS in light of Simon Emmerson's theoretical work (Emmerson, 1986, 2013-14).

Venturing deeper into the aesthetics of sonifications (and sonification-visualizations), we developed a model of perceptual rating scales spanning the APS, and tested it. Following that, we constructed scales to cover qualitative characteristics of interest that are applicable across a range of complex projects that deal with climate data. Through exploratory factor analysis, we identified essential aspects of the projects being studied. Finally, with predictive modeling, we investigated salient relationships between aesthetic dimensions and essential aspects in the dataset.

## 2. Materials and methods

### 2.1. Corpus

Recent years have witnessed an increase in sonification projects that aim to communicate complex, socially relevant phenomena to a larger public (Lenzi, 2021), a transition that the community of data sonification and auditory display advocated for on several occasions. For the present review of projects dedicated to climate change, we have largely followed the PRISMA framework (Preferred Reporting Items for Systematic Reviews and Meta-Analyses, https://www.prisma-statement.org/) to review all the currently listed projects in what is arguably the most complete repository of sonification projects, namely the Data Sonification Archive (DSA; https://sonification.design), a curated, community-sourced online collection launched in 2021, together with works presented at the Conference on Data Art for Climate Action (DACA; http://dataclimate.org) that was held in February 2022. Following the PRISMA flowchart, we initially identified 395 projects: 374 from DSA and 19 from DACA. As this was already a large number for the kind of analysis-intensive study we were preparing, we chose to focus the present study on the complete set of projects listed in these two databases. Future work might start with a broad keyword-matching search on the World Wide Web. As inclusion criteria, we considered projects published within the past 20 years that evidenced a significant component of data sonification relating (in some way or form) to climate action (as in climate change, climate crisis, climate mitigation, and so forth). Projects could additionally employ visualization to represent the data. The first author screened the records and excluded 337 projects (all from DSA; for example, when the title or archival topic indicated that the data was from a source not relevant to climate, such as finance, mobility, astronomy, or war. Another 22 were not eligible because complete information about these projects could not be obtained at the point in time). The selection process yielded a corpus of 32 projects. Of these, 23 are from DSA and 13 from DACA, while 4 appear in both contexts. All focus on sonification of climate data, and 18 out of the 32 projects also include a data visualization component. There are 26 different first authors, out of whom 4 are female, and one whose gender is not known. Including co-authors, there are at least 39 different co-authors, out of which 8 are female, and one whose gender is not known. This imbalance in gender distribution might be looked at more carefully in future studies. The projects are created between 2007 and 2022, with the median year being 2018; this justifies referring to the corpus as consisting of recent research. See Table 1 for an overview of the 32 projects, and Supplementary Data Sheet 1 for details about the corpus, including web links to media and other information.

**Table 1:** Overview of the 32 climate data projects included in the study.

**Project**	**Author(s)**	**Title**	**Year**
p01	Aedes Aegypti	Sonification of atmospheric carbon dioxide in PPM (1958–2008)	2022
p02	Renick Bell and Moon Hung	HKO_hot_temp_rain_sea_1884-2021_20220225	2022
p03	Jon Bellona	#Carbonfeed	2022
p04	András Blazsek	Extreme weather in three movements	2021
p05	Chris Chafe	Hear climate data turned into music	2021
p06	Daniel Crawford and Scott S. George	A song of our warming planet	2021
p07	Enrico Dorigatti	76	2021
p08	Frank Ekeberg	Ingenmannsland	2021
p09	Brian Foo	Too blue	2020
p10	Duncan Geere and Miriam Quick	The natural lottery	2020
p11	Nelson Guda	Treshold	2019
p12	Band of Weeds (Kalle Hamm, Olli Aarni, Lauri Ainala, and Hermanni Keko)	Waiting for the extinction :-(	2019
p13	Band of Weeds (Kalle Hamm, Olli Aarni, Lauri Ainal, and Hermanni Keko)	The weep of trees	2019
p14	Sara Lenzi	While I was not there	2019
p15	PerMagnus Lindborg	Locust wrath	2013
p16	PerMagnus Lindborg	LW24	2015
p17	PerMagnus Lindborg	Stairway to Helheim	2021
p18	Levy Lorenzo	Song of the tides	2018
p19	Duncan Geere, Miriam Quick (Anders Pape Møller)	The end of the road	2017
p20	Falk Morawitz	On the extinction of a species	2017
p21	Hiromi Okumura, Valerie Williams, Jenn Kirby, Thomas B. Jobson, and Joseph Vaughan	Atmos actions	2016
p22	Jamie Perera	Flatline	2016
p23	Jamie Perera	Anthropocene in C major	2015
p24	Jamie Perera	If the oceans could speak	2015
p25	Marty Quinn	The climate symphony	2015
p26	Benjamin Renard	Major flood events	2015
p27	Benjamin Renard and Chloé Le Bescond	Hydrological principal component analysis	2014
p28	Neil Rolnick	Oceans eat cities	2013
p29	Nik Sawe and Lauren Oakes	Sonification of Alaskan forest changes	2013
p30	Katja Striedelmeyer	Shifting apple blossom in bremen—data sonification with a music box	2013
p31	Marco Tedesco and Polar Seeds Group	Polar seeds	2010
p32	Judy Twet	Piano piece	2007

#### 2.1.1. Duration

The duration of project media display (i.e., sonification, visualization) was identified. In several cases, the media display was part of a longer movie (e.g., on YouTube or Vimeo) and only the actual play time of the sonification or visualization was noted. Moreover, the supporting descriptions of projects in many cases included additional audiovisual material, such as spoken presentations or interviews, or sonic output emanating at stages in the design process; these were not counted as part of the media duration.

#### 2.1.2. Lexical diversity

Lexical diversity is one aspect of “lexical richness” and refers to the range of different words used in a text, with a greater range indicating higher diversity (McCarthy and Jarvis, 2010). Different indices exist and many are elaborations on the basic type-token ratio (TTR; the ratio of different unique stems to the total number of words) first developed some 50 years ago. We chose to employ the Measure of Textual Lexical Diversity with moving average window (MTLD-MA; McCarthy and Jarvis, 2010), as implemented in koRpus (Michalke et al., 2021) running in R Core Team (2022).

### 2.2. Content analysis

The corpus was subjected to systematic content analysis. The first author prepared information about each project that included (1) a web link to media (audio-only or movie, i.e., both audio and video); (2) an unformatted text (such as program notes or an abstract); and (3) a web link to other description (such as websites, podcasts, newspaper, and journal papers). All web links were verified at the point of conducting the evaluation (June 2022), and listed in Supplementary material 1. To estimate aesthetic perspective and a range of project characteristics, the 32 projects were evaluated by six researchers, each of whom has skills and knowledge in sonification, visualization, and sonic design. Three are the authors of the present manuscript, two are PhD students with the first and second author, respectively, and one is a research assistant at the first author's lab. They individually evaluated each project according to 33 rating scales which are described further below, in randomized order, *via* an online survey platform (https://www.questionpro.com/). The sonic media itself was considered the most important source for the evaluation, and complemented by text and other descriptions.

#### 2.2.1. Topics

**Figure 1:**
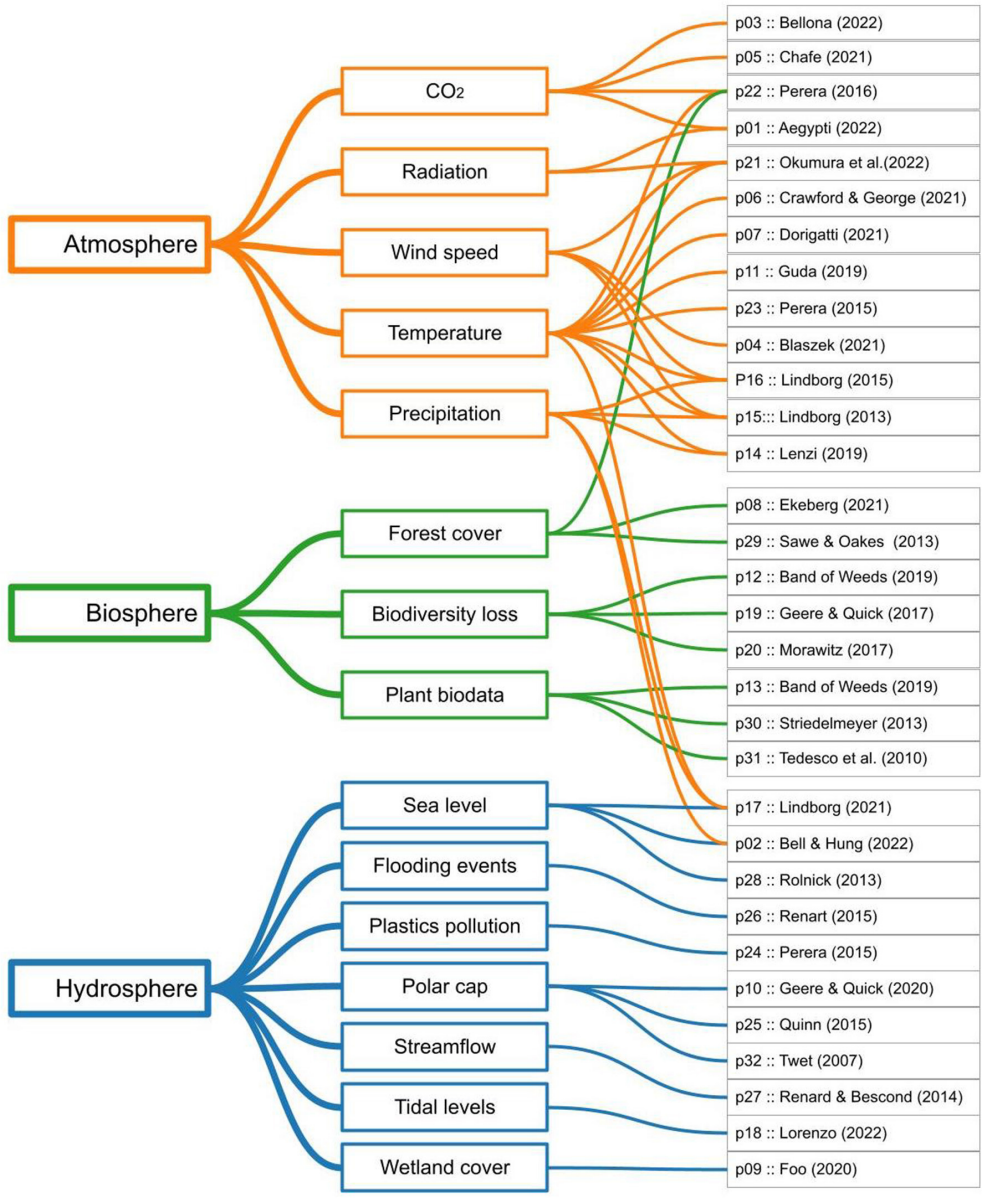
Hierarchical overview of the 32 projects, classified under 3 topics (data provenance) and 15 subtopics (data type).

The project descriptions were of various kinds, containing different amounts of text, images, movie clips, speech, references, and other information, provided by the original authors or by others. Some descriptions were long, such as a published paper of several pages or a substantial webblog, while others were short, such as a program note or artist statement. In 28 out of 32 projects, they were collected from a different site than the media itself. To collate reasonably homogenous textual presentations of the projects, while staying true to the authors' idiosyncratic way of presenting their work, we selected a text-only portion that could be pared down to simple format (ASCII, i.e., no images or HTML). The projects were classified according to topics and subtopics, specifically, the provenance of data (i.e., atmosphere, biosphere, hydrosphere) and the data type (such as CO_2_, Plant biodata, Polar cap etc.). An illustration of the classification is given in Figure 1.

#### 2.2.2. Aesthetic perspective

**Figure 2:**
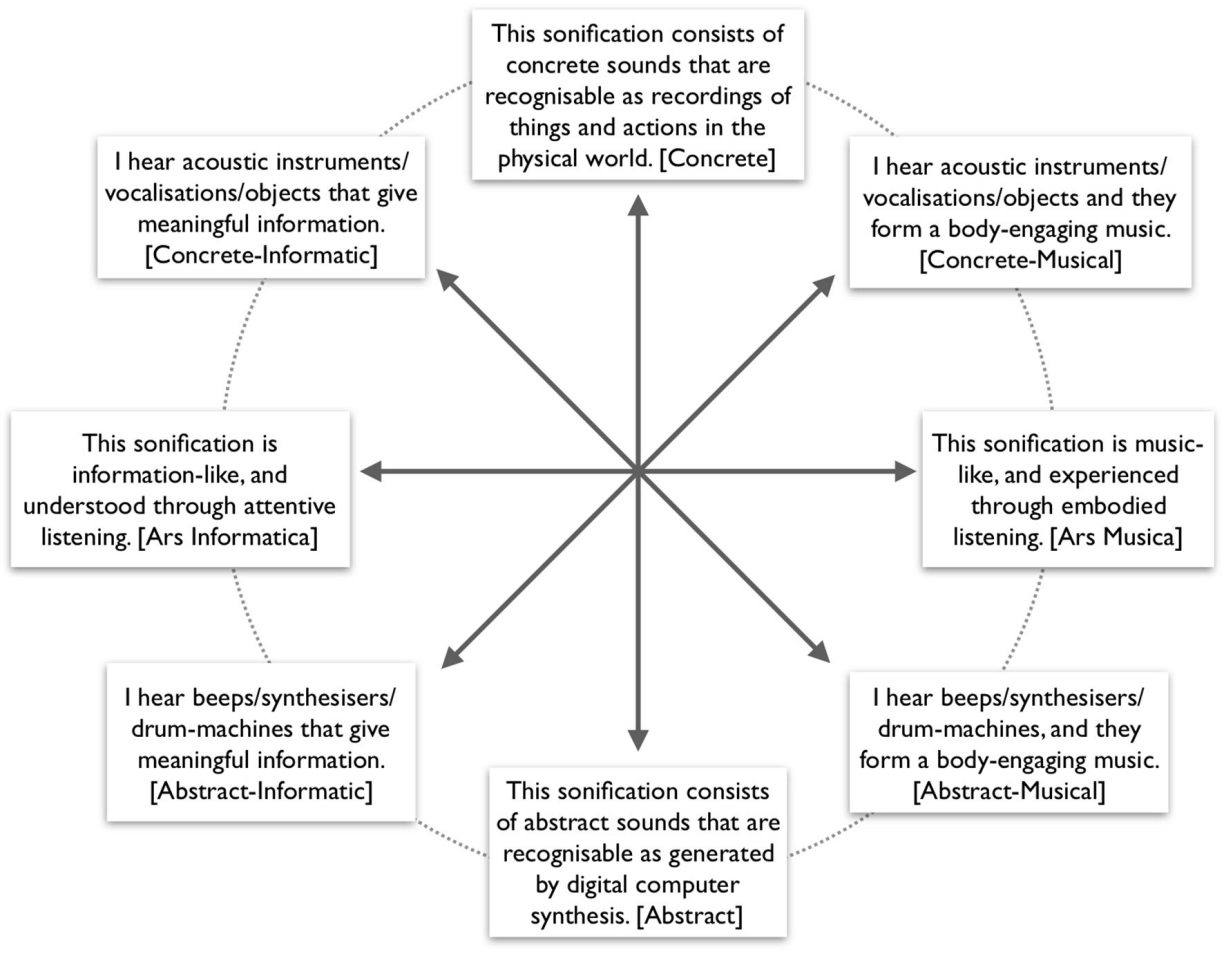
Eight statements of perceived characteristics spanning the Aesthetic Perspective Space.

**Figure 3:**
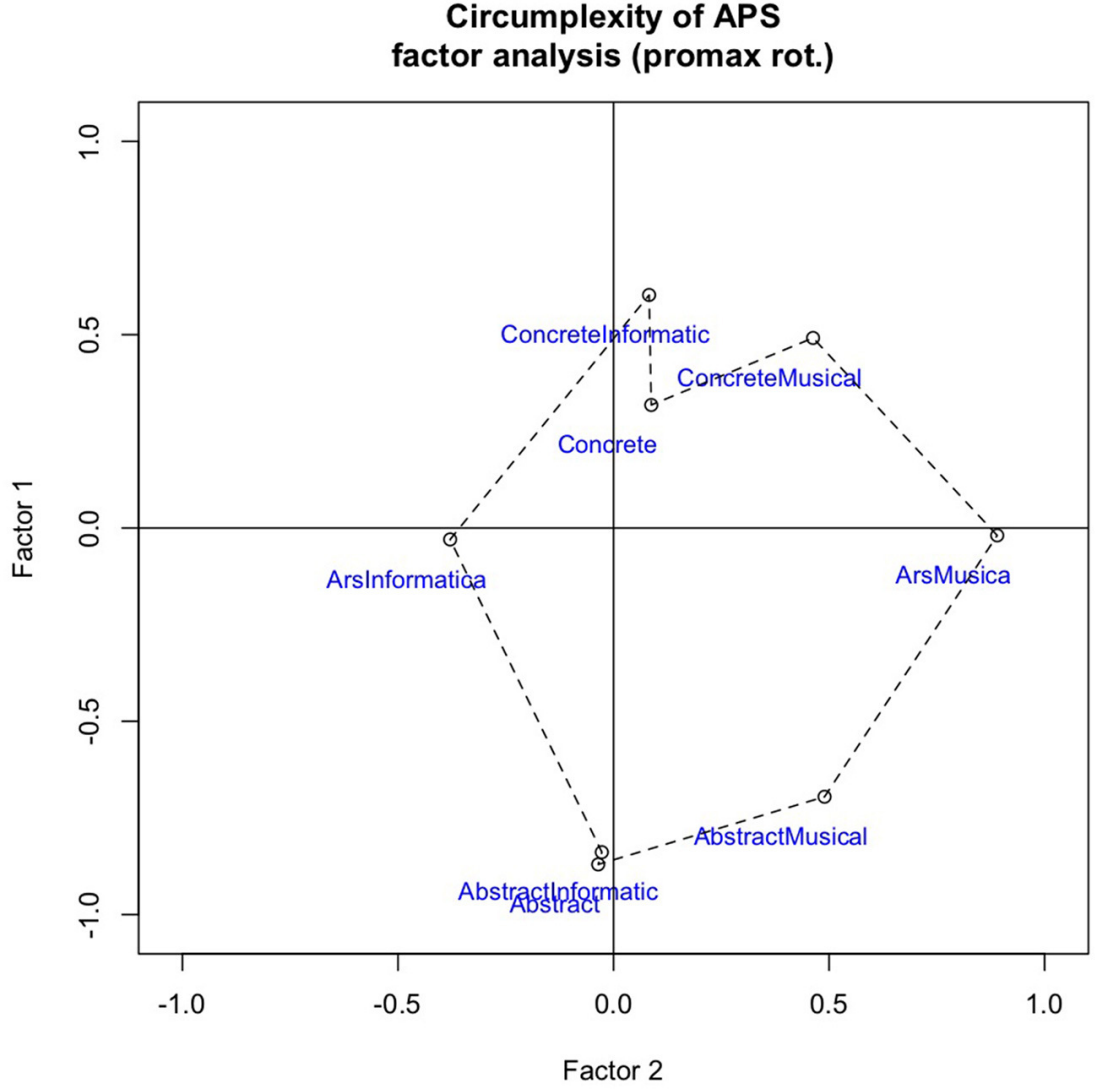
Factor analysis plot to test the circumplexity of eight scales underpinning the Aesthetic Perspective Space.

The Aesthetic Perspective Space (APS; Vickers and Hogg, 2006) has two axes, labeled *Intentionality* and *Indexicality*. We constructed eight unipolar rating scales to span this space, as shown in Figure 2 (compare with Figure 1 in Vickers' article). The circumplexity of this model was evaluated (see Section 3.5 for details). Ratings were made on a seven-step Likert scale anchored by “Strongly disagree” and “Strongly agree,” and the middle marked “Neutral.” Scales were presented in individually randomized order and with left-right direction randomly flipped for each project and rater. The instruction headline was: “Study the text, sounds, and moving images about the project, then globally evaluate how much you agree or disagree, globally, with each of the following broad characteristics.” The word inside brackets is the convenience variable name, used in Figure 3 and in the formulae below (Section 3.4 Aesthetic perspective). See the Discussion (Section 4.1.3) for further details.

- This sonification is music-like, to be experienced *via* listening [Ars Musica].
- I hear acoustic instruments/vocalizations/objects and they form a body-engaging music [Concrete-Musical].
- This sonification consists of concrete sounds that are recognizable as recordings of things and actions in the physical world [Concrete].
- I hear acoustic instruments/vocalizations/objects that give meaningful information [Concrete-Informatic].
- This sonification is information-like, to be understood *via* listening [Ars Informatica].
- I hear beeps/synthesizers/drum-machines that give meaningful information [Abstract-Informatic].
- This sonification consists of abstract sounds that are recognizable as generated by digital computer synthesis [Abstract].
- I hear beeps/synthesizers/drum-machines, and they form a body-engaging music [Abstract-Musical].

#### 2.2.3. Qualitative characteristics

To evaluate a range of characteristics across the projects, 25 rating scales were developed to probe salient aspects of content, methods, and context that would be generally relevant to data sonification and visualization projects. As in the previous part, the researchers who rated the corpus were required to understand the project as a whole before they made their judgements. Ratings were made on seven-step Likert scales labeled “Extremely little”—“Very little”—“Somewhat little”—“Average”—“Somewhat much”—“Very much”—“Extremely much.” Raters were instructed to employ, as far as possible, the full range of each scale across all the projects. The first 20 questions were applicable to all 32 projects, while the last five questions were only applicable to the subset of 18 that integrated data visualization. As before, questions were presented to the raters in randomized order and with left-right direction randomly flipped for each project and rater. The word inside brackets is the convenience variable name which is used in Table 2 and elsewhere in the article.

**Table 2:** Median scores for the 32 surveyed projects.

**ID**	**Intentionality**	**Indexicality**	**Action**	**Technical**	**Context**	**Perspective**	**Visualization**	**logDuration**	**MTLD- MA**
p01	0.03	−4.81	0.20	−0.18	−0.78	−0.34		4.14	83.3
p02	2.93	−4.88	−0.33	−1.48	0.68	0.35	0.58	8.20	22.0
p03	−0.24	−2.19	1.18	0.20	0.75	−0.61	0.44	5.06	117.0
p04	−1.86	−1.35	0.43	0.72	−0.03	0.35		7.66	90.4
p05	−3.88	−4.08	0.29	−1.11	−1.62	−2.28	−0.22	4.53	37.5
p06	−1.96	4.57	−0.65	0.57	0.72	0.26	0.59	4.65	103.4
p07	1.63	3.72	−0.05	0.49	0.31	0.40	0.50	5.46	74.1
p08	0.55	3.34	0.88	0.36	−0.16	0.46		8.20^*^	95.2
p09	−0.05	4.02	0.82	1.15	−0.41	0.00	0.37	4.88	69.5
p10	1.37	−2.60	0.57	0.62	−0.35	0.39		6.30	97.1
p11	−1.24	0.19	0.57	0.01	−0.19	−0.30	NA	2.64	63.6
p12	1.10	−3.80	0.17	−0.88	−0.35	0.43	−1.26	7.03	79.7
p13	−2.73	−2.67	−0.96	−0.58	−0.32	0.31	−1.55	8.20^*^	52.6
p14	0.60	0.79	−0.96	−0.75	−0.69	0.02		7.09	77.0
p15	1.84	−1.93	0.07	1.13	1.30	0.35		7.31	107.0
p16	−2.10	−3.68	0.25	0.68	1.37	0.11		8.01	193.2
p17	0.98	1.99	0.14	0.24	1.23	0.37		7.95	89.4
p18	−2.28	−2.93	−1.41	−0.44	−1.15	−1.28	0.74	4.34	56.4
p19	0.15	−1.50	0.66	0.65	−0.50	0.07		5.86	98.2
p20	1.96	−0.39	−0.01	−1.39	−1.27	0.42		7.23	56.4
p21	−1.69	−2.64	0.07	1.00	1.00	−0.61	0.58	6.53	81.3
p22	1.99	0.37	−0.01	−0.78	1.23	0.55	0.26	5.36	66.8
p23	0.89	3.76	0.68	−0.51	1.00	0.63	−0.67	7.86	79.8
p24	0.93	0.08	0.45	−0.50	0.52	0.49		5.32	94.4
p25	0.61	−1.37	−0.65	1.04	0.23	0.36	−0.73	6.15	59.0
p26	−2.98	2.86	−0.65	0.57	−0.92	−0.91	1.23	3.61	80.2
p27	1.35	2.05	−0.58	0.48	−0.78	−1.01	0.80	4.66	59.5
p28	2.88	3.30	1.10	0.31	1.24	0.37	−0.43	6.89	65.0
p29	0.63	2.93	−0.47	0.01	0.55	1.04		5.16	139.2
p30	−0.49	3.91	−0.05	−0.85	−1.04	−0.69	0.65	3.00	75.0
p31	−1.28	−0.80	−0.66	−0.42	0.06	−0.10	−0.46	4.90	79.4
p32	1.78	3.83	0.66	0.39	−0.08	0.86		5.19	84.4

The derived variables Intentionality and Indexicality span the Aesthetic Perspective Space. The variables Action, Technical, Context, and Perspective are latent factors of the first 20 ratings on qualitative characteristics. The variable Visualization is a latent factor for the last 5 ratings on characteristics that apply when visualization is part of the project. logDuration is the logarithm of duration in seconds; note that p08 and p13, marked with an asterisk (*), are installations of indefinite duration, and for the purposes of the analyses they were set to the maximum value (~1 hour) in the corpus. Finally, MTLD-MA is an index of lexical diversity, where higher values reflect greater diversity. See the text for details.

- How much of the text/description is about the author(s) themselves (as opposed to the work itself)? [Author]
- How much is the text/description about the author's general motivation? [Motivation]
- How much background detail does text/description give about the specific project? [Background]
- How specific is the information about the source data? [SourceData]
- How detailed is the explanation of creative context (such as commissioning body or location of presentation)? [Context]
- How detailed is the recount of impact (such as associated publications, audience testimonies, and visitor numbers)? [Impact]
- How subjective (personal) is the content of the project? [Subjective]
- How objective (distanced) is the content of the project? [Objective]
- How detailed is the information on the original context of fruition (live performance, multimedia product, installation, website…)? [Fruition]
- How detailed is the technical information about the methods of data translation? [Methods]
- What degree of active engagement with the media is called for? [EngageDegree]
- How specific are the instructions for how to engage with the media? [EngageHow]
- How extensive/complete is the legend for understanding how data are represented? [Legend]
- How closely does the media representation match the original phenomenon described by the data? [MatchOrig]
- How convincing is the project in terms of climate science communication? [Convincing]
- How overtly does the project address the climate crisis? [Crisis]
- To what degree is it the author's stated intention for the project to contribute to climate science communication? [SciCom]
- How much does the project raise awareness of the climate crisis? [Awareness]
- How much does the project push for concerted action and adaptation of individual behaviors (e.g., travel, lifestyle choices)?[Behaviors]
- How successful is the project in arousing climate action? [Action]

[If the project includes both sonification and visualization:]

- How important is visualization to the project as a whole? [VisImpo]
- How important is sonification to the project as a whole? [SonImpo]
- In the development of the project, how much did sonification methods drive (initiate) visualization methods? [Son2Vis]
- In the development of the project, how much did visualization methods drive (initiate) sonification methods? [Vis2Son]
- To what degree do visualization and sonification represent the same content? [SonVisConcur]

## 3. Results

### 3.1. Missing values

The individual ratings by the six researchers on 25 scales for 32 projects can be found in Supplementary Data Sheet 3, which is in “long format” and contains 6,336 data points (6 x 33 x 32). There were 118 missing values, which is 1.86% of the total. We can identify two possible causes for missing values. Firstly, the rating process was laborious and took on average 6 h, effectively, to complete. The researchers had to take one or more breaks, and it appears that the QuestionPro software did not always register the last few ratings before the responses were saved in their system. Secondly, the web server for one project (p31) was unavailable to two raters, who thereby had to skip 66 ratings. All missing values were imputed with the median of within-project ratings by the others. The post-processed data are given in Supplementary Data Sheet 4 (together with computed variables; see below). A conveniently compact layout of median ratings is offered in Table 2.

### 3.2. Duration

The duration of project media (i.e., sonification, visualization) was approximately exponentially distributed. The median duration was just under 4 min, in a range from 14 s to about 1 h. There were two installation-type works having indefinite duration (p01 and p13) and for the purposes of the analyses their durations were set to the maximum value of the longest specified media duration in the corpus. We defined a variable logDuration as the natural logarithm of the media duration in seconds; this variable was normally distributed (Shapiro's *W* = 0.95, *p* = 0.14).

### 3.3. Lexical diversity

The descriptive texts contained on average 465 words each, in a range from 52 to 1,533. While this is a large range in text volume, note that a study by McCarthy and Jarvis (2010) showed that MTLD-MA was the only index not varying as a function of text length. In our analysis we adopted the default setting for the TTR factor size of 0.72. In the present data, lexical diversity (MTLD-MA) had a positive skew (*W* = 0.88, *p* < 0.001); this is partly due to a very high value for one project (p16, for which the descriptive text had been extracted from a peer-reviewed journal article). The scores for logDuration and lexical diversity are listed in Table 2 further below.

### 3.4. Inter-rater agreement

The six researchers individually evaluated the 32 projects, taking from 5.5 to almost 7 h to complete the ratings over several sessions. That is, they spent around 12 min per project. The interrater agreement was good, as indicated by Cronbach's alpha = 0.80 across the 33 scales.

### 3.5. Circumplexity of APS

As explained above (Section 2.2.2 Aesthetic perspective), the first eight question-scales were aimed at capturing the two main dimensions, labeled Intentionality and Indexicality, in the Aesthetic Perspective Space [APS]. To test if the APS circumplex (specifically, a circulant) would be an accurate representation of the current data, we followed the procedure outlined by Tracey (2000), also considering Acton and Revelle (2004). A circulant is defined by equal spacing of variables around a circle. Testing proceeded in three steps: (1) “eyeballing” factor analysis plots; (2) analyzing the residual matrix (Hartmann et al., 2018); and (3) conducting tests of the circulant hypothesis, i.e., equal spacing of variables along the circle and equal radii (loading strength of the eight variables onto two latent factors corresponding to the two main factors of the APS. We used a bootstrap method to estimate the probability of the observed data appearing spuriously.

Firstly, inspection of the biplot in Figure 3 supports an intuitive understanding of the eight variables as forming a roughly circular shape. Note that rotation of factors does not change the evaluation of circumplexity. To make the “comparison-by-eyeballing” straightforward, we have rotated the plot by swapping the two factors between x and y axes, and then flipping the y-axis. This makes the plot of our current observed data more resemblant to Figure 2, which illustrates the theoretical model. The most important distortions are for AbstractInformatic, which loads too close to abstract (i.e., highly correlated), and ConcreteInformatic, which loads close to Concrete. The reasons for this might be found in somewhat differing understandings of the rating scales among the six researchers. Despite good inter-rater agreement (Cronbach's alpha = 0.83 for the eight APS scales), the raters might have reacted in subtly different ways that are not captured by the alpha statistic. Inspecting individual plots (such as Figure 3), we could observe that one researcher produced an almost perfect circle, two had shapes very similar to the average, one had a shape that was slightly more distorted, and the shapes of the last two were more non-circular. Nevertheless, we decided to keep all the six raters.

Secondly, analyzing the residual matrix for all the data, we found that the two indicators given by Hartmann et al. (2018) supported the assumption that our factor model was a good representation of the underlying concept, i.e., correspondence to the two main dimensions of APS. After fitting our data to the theoretical model, the off-axis values in the residual matrix were “close to zero” for each of the eight factors (mean = 0.026), and the maximum (0.15) was well within the range indicated by Hartmann.

Thirdly, we followed Tracey (2000) to evaluate the equal distribution of factors along the rim of the circle. In the data, the gaps (or Distance-to-Next, as in Acton and Revelle, 2004) between observed factors and their theoretical position were {−1, 2, −15, −53, 4, 43, −2, −10} degrees, counting from ArsMusica counter-clockwise to AbstractMusical. Note that the highest absolute values are for ConcreteInformatic (−53) and AbstractInformatic (43), confirming what we eyeballed previously. The radii were calculated as the Euclidean distance of loadings from the center. To create bootstrap distributions for angles and radii, 10,000 uniformly randomized sets of 192 pseudo-ratings were generated. The variance of angular gaps and variance of distance-from-center scaled by mean distance were calculated (Tracey, 2000). Comparing the lower end of the sorted distributions with the values for our current data yielded probabilities for the observed data to occur spuriously. They were: *p* ≤ 0.000^***^ for even distribution of factors along the rim of the circle, and *p* = 0.0102^**^ for the radii being similar in length. The two formal criteria for the assumption of circumplexity (circulant) were thus met in the current data. We proceeded by calculating the position for each project and rater according to the theoretical model of the Aesthetic Perspective Space from the ratings on eight scales as follows:


$$\begin{array}{r} \;Intentionality=ArsMusica-ArsInformatica+\\ \sqrt{2}/2^{*}(ConcreteMusical-Abstract\;Informative+\\ AbstractMusical-ConcreteInformatic)\\ Indexicality=Concrete-Abstract+\\ \sqrt{2}/2^{*}(ConcreteMusical-AbstractInformatica+\\ ConcreteInformatic-AbstractMusical) \end{array}$$

**Figure 4:**
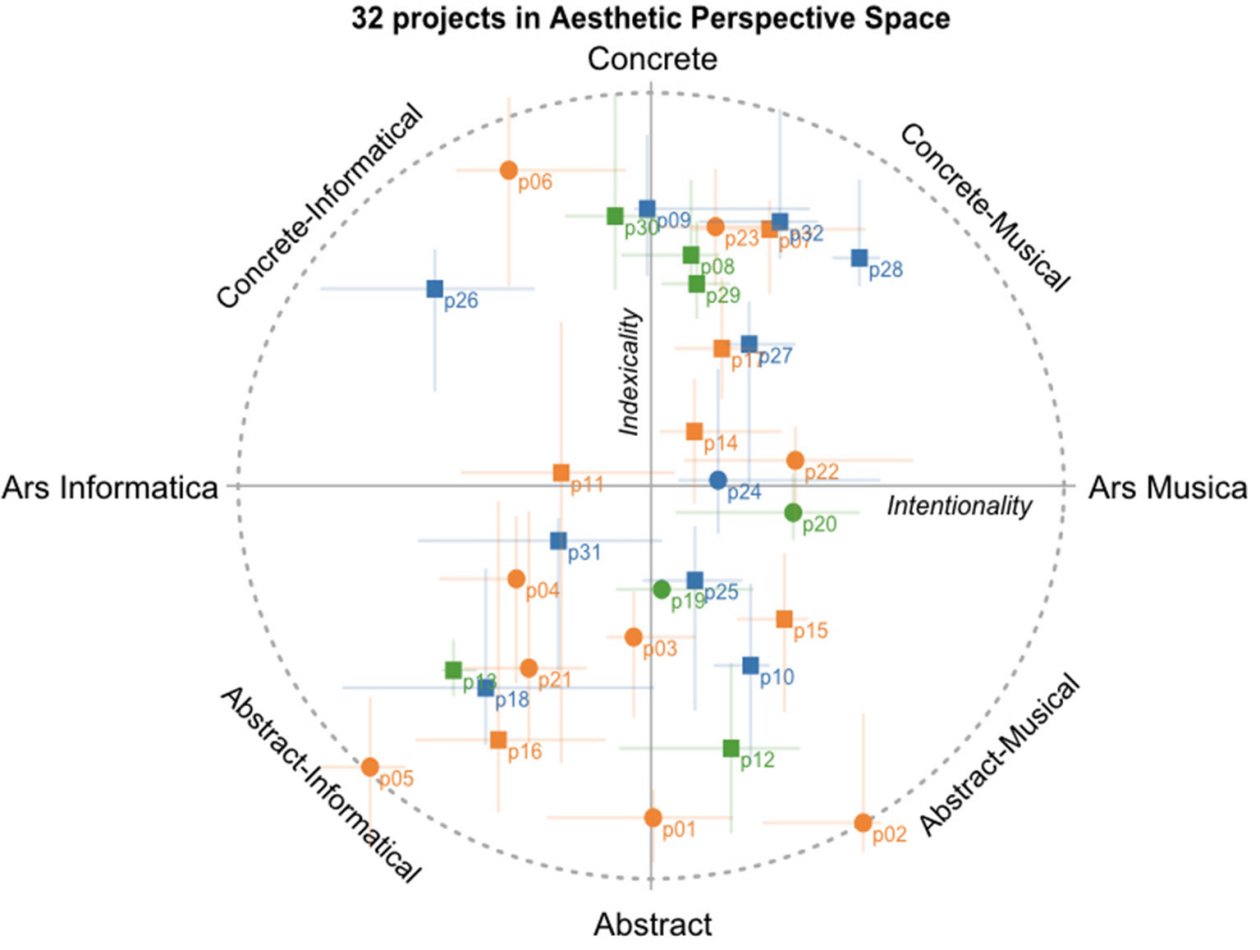
Mapping of 32 projects in the Aesthetic Perspective Space: median position with lines indicating the 1st and 3rd quantiles. Circles, sonification projects; Squares, sonification-visualization projects. Colors denote topics (data provenance) as follows: orange, atmosphere, green, biosphere, blue, hydrosphere.

While the distribution of Intentionality was normal (Shapiro's *W* = 0.97, *p* = 0.14), that of Indexicality did not pass the test (*W* = 0.91, *p* = 0.014). Pearson's measure of kurtosis was −1.51, as calculated with the psych package (Revelle, 2020) running in R Core Team (2022), indicating a thin-tailed distribution. If true, the presence of a broad or even bimodal distribution (a positive and a negative node) might indicate that the raters dichotomized amongst the projects along the Indexicality dimension, and tended to make a categorical distinction between abstract and concrete sonic materials. The matter might be addressed in a future study that considers details of causal listening that pertain to action-sound couplings (Tuuri and Eerola, 2012; see Lindborg, 2019 for a proposition). For the present analysis, it is important to note that one assumption for the validity of linear regression results is that residuals of the fitted dependent variable are normally distributed, but it is not required that the variable itself is normal. Nevertheless, we proceed with some caution in interpreting results that involve Indexicality. The scores (medians across raters) are listed in Table 2 further down, and illustrated in Figure 4. Note that the variance of Intentionality was about half that of Indexicality, so that the distribution visually appears somewhat “squashed.” The aesthetic perspectives of several individual projects are discussed below, in Section 4.2.

### 3.6. Qualitative characteristics

The 25 rating scales for qualitative characteristics were developed with the assumption that they would cover (to some degree) essential aspects across the 32 projects. These essential aspects can be understood as latent factors in the data, and exploratory factor analysis provides an estimate of the correlations with the latent factor(s) representing the data Revelle (In Press). In the present analysis, the number of factors was determined with the nfactors function in the psych package (Revelle, 2020) running in R Core Team (2022). We evaluated the functions output of VSS Complexity (VSS; Very Simple Structure) and Empirical BIC (Bayesian Information Criterion) to determine the optimal number of interpretable factors (Revelle and Rocklin, 1979). We then used the fa function from the same library, with settings for ordinary least squares regression and promax rotation, to find a minimum residual solution with factors that lent themselves to a straightforward interpretation in terms of essential aspects of the projects.

In the first analysis we included all the 32 projects and the first 20 rating scales. A parsimonious solution was found with four factors, which together explain 56% of the variance in the data (32 projects × 20 scales). They were labeled Action, Technical, Perspective, and Context. We proceeded by analyzing the 18 projects that integrated both sonification and visualization but this time only including the 5 rating scales applicable to them. A solution was found with one factor, labeled Visualization, that explains 39% of the variance in this subset of the data (18 projects × 5 scales). The latent factors and their loadings on rating scales are given in Table 3, and discussed further below.

**Table 3:** Exploratory factor analysis of ratings of qualitative characteristics in 32 projects.

**Rating scale**	**Action**	**Technical**	**Context**	**Perspective**	**Visualization**
Author					
Motivation		0.431		0.44	
Background		**0.757**			
Source data		**0.854**			
Context			**0.826**		
Impact			**0.804**		
Subjective				**0.786**	
Objective				−0.69	
Fruition			**0.821**		
Methods		**0.841**			
EngageDegree	0.453				
EngageHow		0.42			
Legend		**0.897**			
MatchOrig					
Convincing	**0.789**				
Crisis	0.634				
SciCom	0.483				
Awareness	**0.947**				
Behaviors	**0.717**				
Action	**0.907**				
**Variance Prop**.	0.192	0.169	0.113	0.079	
**Variance Cum**.	0.192	0.366	0.479	0.559	
VisImpo					**0.874**
SonImpo					
Son2Vis					
Vis2Son					**0.77**
SonVisConcur					**0.751**
Variance Prop.					0.389

For clarity, loadings <0.3 have been removed, and loadings >0.7 are in bold. Note that two separate and non-overlapping analyses were made on the data; see text for details.

The loadings of the individual rating scale variables onto the latent factors can be gathered by inspecting Table 3. We may note that the first and relatively strongest factor, labeled Action, is influenced foremost by ratings on the scales named Awareness, Action, Convincing, and Behaviors. Similarly, the second factor, Technical, is determined by Legend, SourceData, Methods, and Background, while the third, Context, by the rating variables named Context, Fruition, and Impact. Finally, the fourth is positively influenced by Subjective and negatively by Objective. The last of the latent factors, Visualization, determined by a separate analysis of the subset of 18 projects that integrated visualization, was positively influenced by VisImpo, Vis2Son, and SonVisConcur. To revise the constructs of the 25 rating scales, see the exact wordings for the questions posed to the raters; they are listed in the section above (Qualitative characteristics).

### 3.7. Multivariate analysis

We then investigated the relationship between Intentionality and Indexicality (APS dimensions) and the essential characteristics (latent factors), namely Action, Technical, Context, Perspective, and Visualization, together with logDuration and MTLD-MA. As in the exploratory factor analysis, we conducted two separate analyses: one on the whole dataset of 32 projects (20 rating scales yielding 4 latent factors), and the other on the subset of 18 projects that included visualization (5 rating scales yielding one latent factor). In this analysis, the ratings were z-scaled (“standardized”) within each rater. Firstly, we tested for multivariate relationships with MANOVA, taking Intentionality and Indexicality as jointly dependent variables, including as the independent variables the factors Action, Technical, Context, and Perspective (in the first case), or Visualization (in the second case), as well as logDuration and MTLD-MA. In both cases, the multivariate analysis of variance revealed the presence of significant differences. We therefore proceeded with modeling the univariate relationships with linear regressions, taking in turn Intentionality and Indexicality as the dependent variable (predictand) and the same variables listed above as predictors. The results are listed in Table 4. We tested the validity of these results by analyzing the residuals of the dependent variable in each of the four models. In the first case, the residuals for Intentionality after model fitting were near normal (Shapiro-Wilk's *W* = 0.98, *p* = 0.01) and passed the test for heteroscedasticity (Breusch-Pagan's BP = 4.5, *p* = 0.35). Meanwhile, Indexicality residuals were normal (*W* = 0.99, *p* = 0.09) and heteroscedasticity was not present (BP = 12.9, *p* = 0.11). In the second case, for projects involving data Visualization, residuals passed the two tests both for Intentionality *W* = 0.98, *p* = 0.04, BP = 6.8, *p* = 0.08) and for Indexicality (*W* = 0.98, *p* = 0.11, BP = 13.4, *p* = 0.04).

**Table 4:** Statistics for regression models predicting Intentionality and Indexicality from rated characteristics, duration, and lexical diversity, in all 32 projects and a subset of 18 projects integrating visualization.

	**Intentionality (R2** = **0.27, adjusted** = **0.25)**				**Indexicality (R2** = **0.17, adjusted** = **0.14)**			
**In 32 projects**	**est**.	***t***	***p***	**ß**	**est**.	***t***	***p***	**ß**
Action	0.38	2.48	0.014^**^	0.16	0.09	0.36	0.72	0.02
Technical	−0.33	−1.87	0.062.	−0.13	0.33	1.18	0.24	0.09
Perspective	1.19	6.32	0.000^***^	0.48	1.44	4.86	0.000^***^	0.39
Context	0.18	1.00	0.32	0.07	0.24	0.83	0.41	0.06
logDuration	0.02	0.22	0.83	0.02	−0.62	−3.84	0.000^***^	−0.29
MTLD-MA	−0.01	−2.68	0.008^***^	−0.19	0.00	−0.44	0.66	−0.03
	**Intentionality (R2** = **0.12, adjusted** = **0.10)**				**Indexicality (R2** = **0.15, adjusted** = **0.12)**			
**In 18 projects**	**est**.	***t***	***p***	**ß**	**est**.	***t***	***p***	**ß**
Visualization	0.49	1.84	0.068.	0.20	0.41	1.10	0.28	0.11
logDuration	0.66	3.82	0.000^***^	0.42	−0.34	−1.39	0.17	−0.15
MTLD-MA	0.01	0.64	0.52	0.065	0.04	2.85	0.005^***^	0.28

All intercepts were non-significant and have been removed for clarity. R2, amount of total variance explained; adj., R2 adjusted for the number of predictors; est., estimated coefficient for the variable. t, coefficient divided by its standard error; p, probability value, with asterisk codes for degree of significance: ^***^p < 0.001; ^**^p < 0.01; ^*^p < 0.05; ß, standardized beta coefficient.

**Figure 5:**
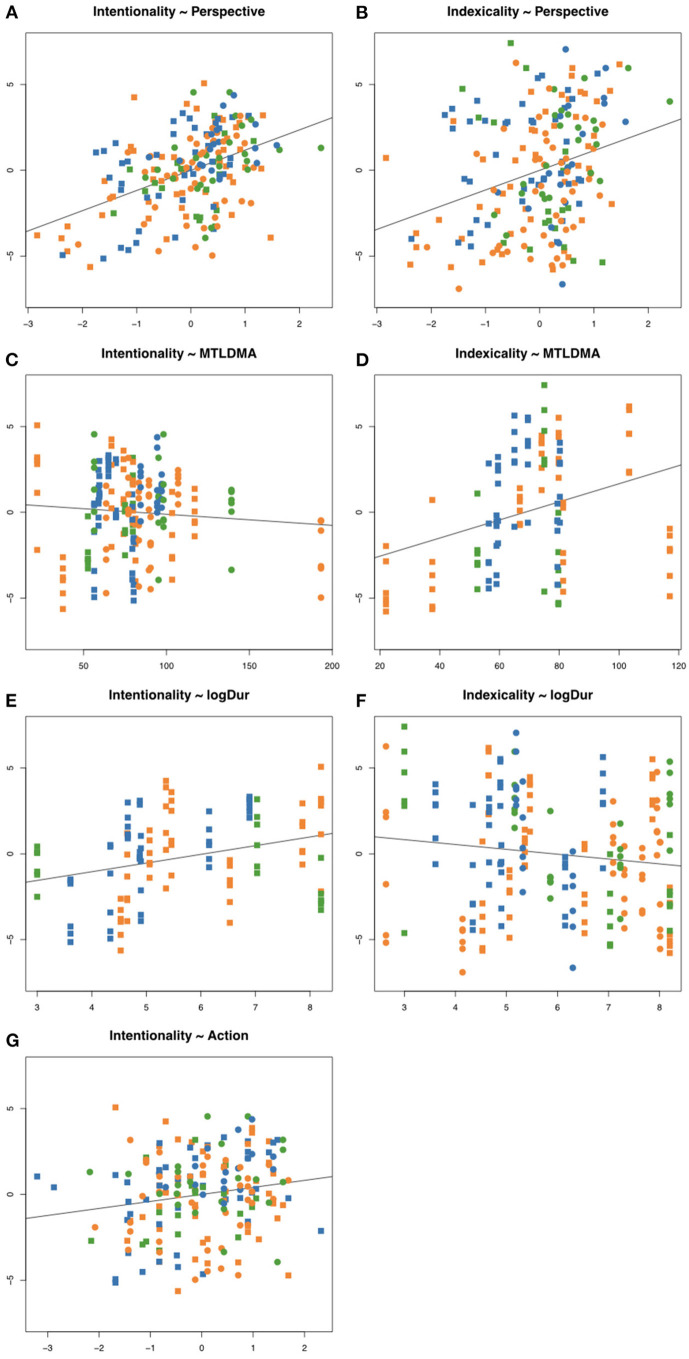
Scatterplots of significant relationships in regression modeling. In all Panels, units for outcome variables (predictands) are the centered ratings of Intentionality (left column) and Indexicality (right column), derived from the eight scales spanning the Aesthetic Perspective Space. In each of Panels **(A, B, G)**, the predictors are the latent factors Perspective and Action, respectively, in the original units from ratings scales, and centered. In Panels **(C, D)**, the predictor is MTLDMA (lexical diversity), in original units (see section 2.1.2 for details). In Panels **(E, F)**, the predictor is duration (logarithmic; see section 2.1.1 for details). Shape and color of symbols are the same as in Figure 4.

To explore the models further, we applied stepwise reduction, but this did not yield additional information worthy of the effort. Since there are relatively few predictors involved, we believe it is more useful for comparisons to study the regression results while keeping the same set of predictors. With this in mind, we offer an interpretation of results listed in Table 4. For a visual illustration of the seven significant relationships in the data, see Figures 5A–G.

For Intentionality, the significant predictors were the latent variables Perspective (ß = 0.48; Figure 5A) and Action (ß = 0.16; Figure 5G), together with the lexical diversity measure MTLD-MA (ß = −0.19; Figure 5C); within the subset of projects involving visualization, logDuration was a significant predictor (ß = 0.42; Figure 5E). In other words, the perceived “Ars Informatica vs. Ars Musica” dimension in sonifications was associated with the perspective of “objective vs. subjective” gleaned from the written descriptions. Projects that tended toward “Ars Musica” were described in simpler language yet produced more convincing characteristics that emphasized awareness, action, and change of behavior. When visualization was an integral part, “Ars Musica” projects were longer in duration.

For Indexicality, the significant predictors were Perspective (ß = 0.39; Figure 5B) and logDuration (ß −0.29; Figure 5F), and within the subset of projects involving visualization, also lexical diversity (ß = 0.28; Figure 5D). In other words, the “Concrete vs. Abstract dimension,” which refers to the perception of sonic materials, was again associated with the perspective of “objective vs. subjective” in written descriptions. Sonifications with more concrete sonic materials (such as recognizable acoustic instruments or samples of natural sound sources) were longer in duration, and within the subset of projects that involved visualization, were described using simpler language.

## 4. Discussion

### 4.1. Results

#### 4.1.1. Regression models

From the tables and scatter plots, we may identify and highlight relationships that the statistical analysis has revealed. We see that Perspective was a strong positive predictor for both Intentionality and Indexicality, even though the two dimensions were not significantly correlated (Spearman's rho = 0.16, *p* = 0.38, calculated on medians across raters). Recall that a high value on the latent factor labeled Perspective is mainly due to high ratings on the scale named Subjective, and low on the Objective scale. Projects with more author-oriented descriptions thus predicted a sonification output in the Concrete-Musical quadrant (in our corpus, p28 “Oceans Eat Cities” by Rolnick is the clearest example of this; see Discussion below for details). Across the corpus, duration (logarithm) was a negative predictor of Indexicality, so that longer sonifications were generally more abstract in terms of their sonic materials (as exemplified by p12 by Hamm and p16 by Lindborg; however, not in p01 by Aegypti). Higher lexical diversity, measured by MTLD-MA, predicted a lower value for Intentionality, i.e., sonifications perceived as Ars Informatica (p05 by Chafe and p26 by Renard are clearly science-oriented projects).

At the same time within the subset of 18 projects integrating visualization, higher lexical diversity predicted higher values of Indexicality, indicating that richer textual descriptions were associated with concrete rather than abstract sonic materials (e.g., p09 by Foo, which features marching band music). Similarly, logDuration was strongly positively associated with Intentionality, which is to say that visualization-sonification projects perceived as Ars Informatica were shorter (e.g., 26 by Renard). Note that these two effects were not significant when all 32 projects were taken under one, which might indicate that, across the whole corpus, sonification-only projects countered them: shorter projects might well be Ars Musica (e.g., p22 by Perera), and richer descriptions might indicate abstract sounds (e.g., p01 by Aegypti).

#### 4.1.2. Topics and characteristics

As we read the project descriptions very closely to determine topics and subtopics, many other types of questions came to mind. For example: How rich or multidimensional is the source data? How large is the data set(s) being referred to? Are the data sets referred to available in the public domain? From the information given, to what extent is the project replicable? Such questions eventually boiled down to the 25 qualitative characteristics employed in the scale ratings, which generated the five essential aspects used in the analysis. In future work that attempts to further explore the notion of characteristics of complex projects, we would recommend a “minimalist approach” and develop a compact protocol for aesthetics (probably eight scales) and characteristics (for example 10 scales, i.e., the five essential aspects from our present findings, paired with “reverse coded” questions). The number of potential qualitative characteristics (i.e., qualia) of complex projects is quasi infinite. The set of 25 represented a compromise between the wish to cover as much terrain as possible, and a need to keep the time demanded of the raters reasonable. It still takes many hours to complete a full set of ratings. In future work, we will look into ways of speeding up the process.

**Figure 6:**
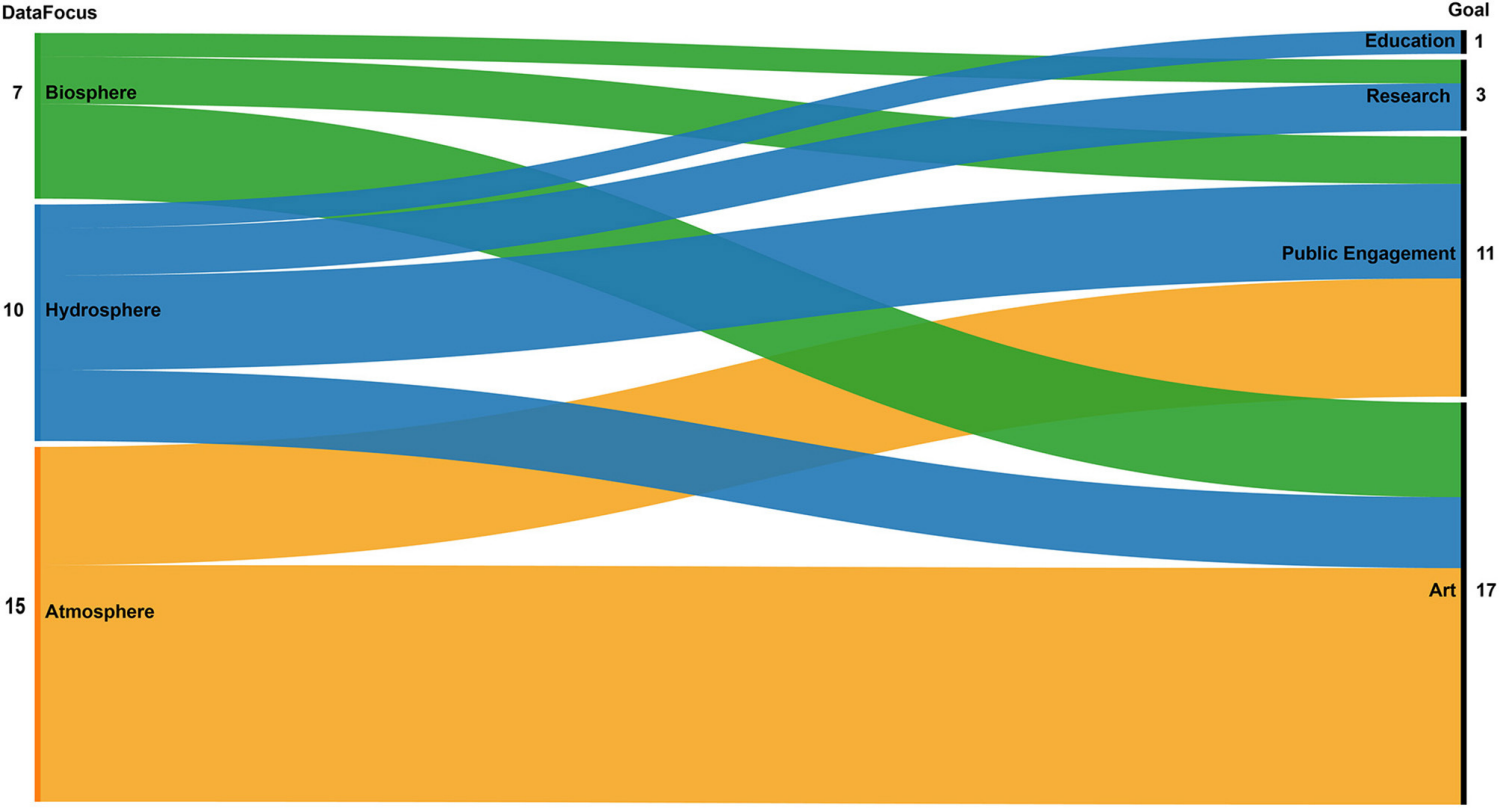
Correlation between the three data foci (atmosphere, biosphere, and hydrosphere) and the goals of the selected projects.

A closer look at the relationship between the topics emerged from the analysis (see Figure 6, Atmosphere, Biosphere, and Hydrosphere: in orange, green, and blue, respectively) and the other properties used in the classification of cases, borrowed from the metadata classification protocol of the DSA, shows that only one case that focuses on data of the Hydrosphere was created for educational purposes and the same topic along with biospheric data was the focus of research projects. Cases that used atmospheric data represent the biggest group that has art and public engagement as the main goals.

**Figure 7:**
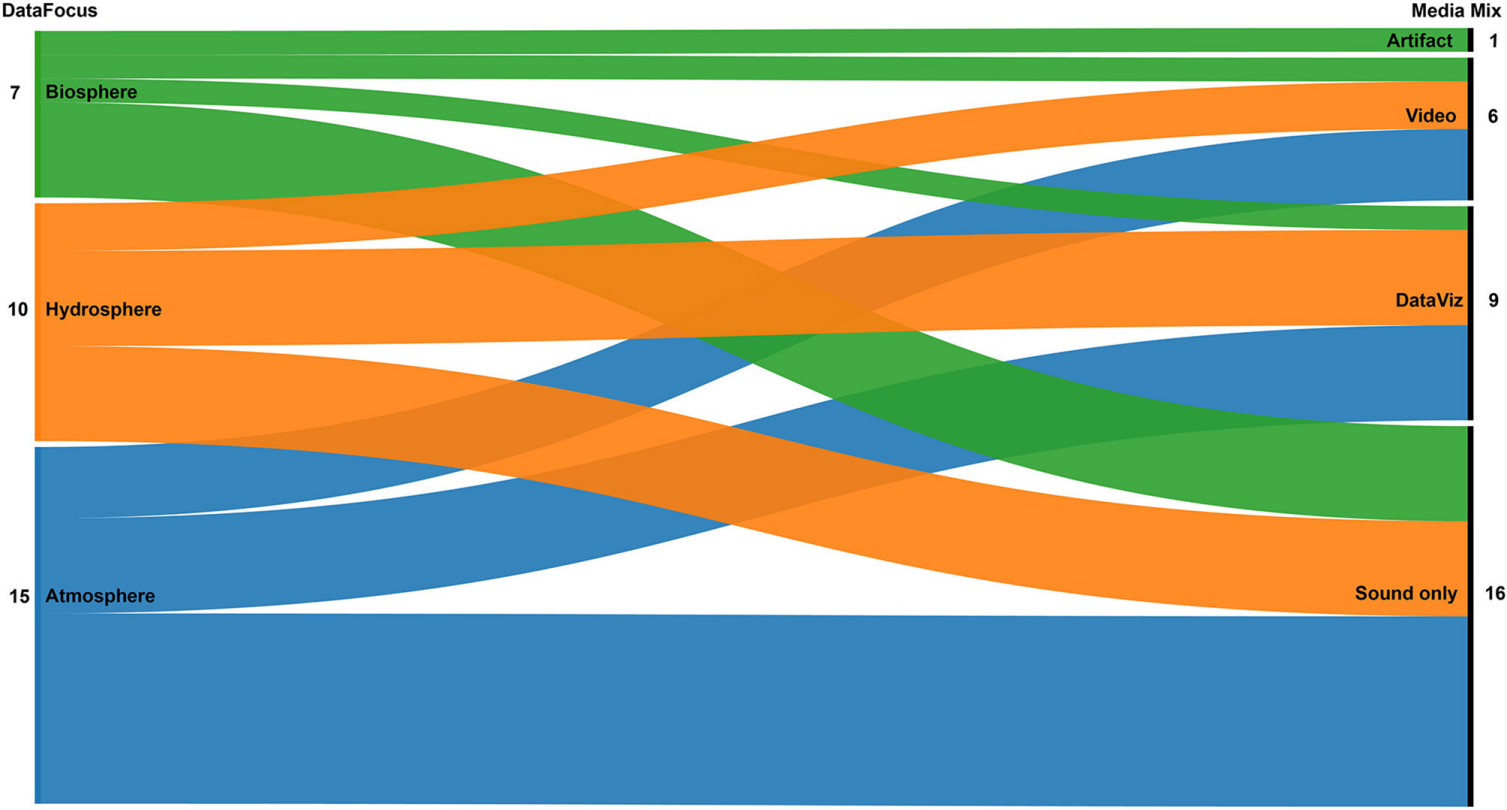
Correlation between the three data foci (atmosphere, biosphere, and hydrosphere) and the mix of media used in the selected projects.

Figure 7 illustrates correspondences between the three emerging topics and the media mix. Media mix is a specific metadata used in the DSA to classify projects by the medium used along with sound. Examples of media mix are “Sound only” (i.e., when the project only uses sonification), “Data viz” (i.e., when sonification and data visualization are combined), “Video” (i.e., sonification is used in combination with visual content in form of moving images), and “Artifact” (i.e., the sonification is created by interacting with a tangible object). In general, the three emerging topics (i.e., “Data Focus”) use a good mixture of media to represent data. Phenomena related to Biosphere and Atmosphere are mainly represented using sound only, while Hydrosphere datasets are evenly using sound alone and sound combined with data visualizations. Video content that does not replicate the sonified data, rather is used as a support for engagement, is used for all the topics. The only project that uses a physical, automatic artifact (p30; specifically, a music box, documented in a movie clip) to generate the sonification is found in the Biosphere group.

#### 4.1.3. Aesthetic perspective space

The primary (horizontal) axis in the Aesthetic Perspective Space, Intentionality, is based on the theoretical proposition that there exists a continuous, bipolar, conceptual, psychological mechanism accessible by an intentional mode of listening that someone might apply when presented with an auditory object. The listener perceives the designed intention behind the object, and interprets its communicative focus on a scale between information-extraction and artfulness-experience. The secondary (vertical) dimension maps the perceived qualities of the sonic materials that the object is constituted by and their ontology, that is, whether the listener perceives the object as abstract-imaginary or as concrete-physical. This characterization of the sonic source material leans on Simon Emmerson's Language Grid, a framework that affords an analysis of electroacoustic music along two continuous dimensions: one describing the composer's perceptual attitude to the musical material, from Aural to Mimetic, and the other describing the composer's action on the material, from Phonographic to Constructed (Emmerson, 1986; Fischman, 2007; also and importantly, Emmerson, 2013-14). Emmerson defines “mimetic” as “the imitation not only of nature but also of aspects of human culture not usually associated directly with musical material.” (Emmerson, 1986, p. 17). Within the context of auditory display, Vickers identifies sonification as a form of “mimetic discourse,” where “indexical” appears to be exactly the same as “mimetical.” One might therefore speak of “listening to concrete mimesis' in a situation where a sound object unequivocally denotes a physical source that is present in the environment,” and “listening to abstract mimesis” when a sound object associates with a non-present source or concept through metaphor. The association might be more or less graspable, hypothetical, or private.

We believe that the APS, as a conceptual tool, is very useful for research in audio design and auditory perception of both music and sonification: it merits thorough testing and further development. In their article presenting the Aesthetic Perspective Space, Vickers and Hogg (2006) had populated the circumplex with two handfuls of examples: some were specific pieces, others were generic, such as a genre or a composer. In subsequent papers, Vickers (2016); see also Vickers et al. (2017) included weblinks for most of the examples. It is not clear whether the positioning of these example works were empirical or hypothetical. Our present study is a step in a larger project of testing the underpinnings of APS, as a theoretical proposition, against empirical observations. We have used methods (e.g., corpora, experimental procedures, rating scales) that are replicable and extendable by other researchers. For example, it should be feasible to conduct a listening test to explore how people interpret a larger corpus of shorter clips that contain different types of sonic artifacts: composed EAM pieces, sound art, soundscape recordings, and data sonifications. A qualitative analysis of interviews with the test subjects might provide insights into their evaluation strategies (as in Lindborg and Friberg, 2015).

### 4.2. Observations of projects

Our analytical approach has been informed by the ongoing discussion in sound design and auditory perception research communities on the relationship between sonification and electroacoustic music composition. The authors are part of these fields in various ways. The first author is the main organizer of the DACA festival and a research-driven composer with a focus on multimedia experiences. The second author is active in the area of sound-driven design research and data sonification. She is the co-curator of the DSA and an evangelist of the potential of data sonification amid the information design and data visualization community. We will deepen this notion of “relationship” by giving examples of qualitative observations of a few of the corpus projects.

Looking at the projects with highest and lowest scores for Intentionality and Indexicality provides insights into the characteristics that create aesthetic perceptions: such as, the most “musical” or “informatics” sonification, or the one having the most “concrete” or “abstract” sonic materials.

The project that scored highest on Indexicality (concrete sound materials) was “Shifting Apple Blossom in Bremen” (p30) by Striedelmeyer. It stands out because data are sonified (as well as visualized) through a physical music box which the user (e.g., at an exhibition) would manually activate in order to hear the data.

The two projects that scored highest on Intentionality (“Ars Musica”) were both by Jamie Perera. A personal communication between the second author and Perera clarified that the composer's interest in sonification lies mainly in the potential of this translation method to increase public engagement on critical topics (such as climate change, but also the COVID-19 pandemic), support activism and overall take responsibility as artists toward society at large.

The 18 projects that integrate data visualization with sonification are diverse. With “Anthropocene in C Major” (p23), Pereira has composed a 45-min orchestral piece, accompanied by a visualization of the dataset so as to work as a performance guide for the public. Pereira released six sonification projects between 2017 and 2020, and three are included in the corpus (p22, p23, p24). Each project highlights a different consequence of climate change.

Some of the reviewed projects materialize as pieces with multiple movements or sequential parts. When the movements use different techniques and aesthetic style it becomes hard to evaluate the project as a whole. For example, Blazsek's project “Mongkut” (p04) was the sonification most clearly identified as Ars Informatica (see Figure 4 and Table 2). The work has three movements where the first and last use similar techniques and style (e.g., sinewave modulation) while the middle movement displays a very different sonic characteristic (e.g., concrete soundscape recordings). The durations of movements are widely different (short, very long, very short). This compositional diversity of approach poses challenges for the raters to judge the characteristics and thus to pinpoint the project as a whole. The fact that this project draws on the same event/phenomenon does hold things together as a scientific demonstration, and as the Intentionality score indicates, lends it to an appreciation as utilitarian rather than experiential.

Ekeberg's sonification installation “Ingenmansland” (p08) shows some similarities with p04. Here, there are two movements where the musical textures are similar though the sonic materials are distinctly opposite (first concrete, then abstract). As in Blazsek's tripartite piece, Ekeberg presents a diptych whose parts are held together since they refer to the same subject, deforestation in western Norway: first as a pseudo-documentary field recording, then as an electroacoustic, dystopian metaphor.

By contrast, in “Oceans Eat Cities” (p28), Rolnick lets the two movements have differing musical style (e.g., tempo, density, character) yet the characteristics of the sonic material remain the same throughout. Apparently, the way the data was used to determine musical materials is consistent across the movements of the piece.

Some project descriptions didactically present stages in a process, such as in “Polarseeds” (p31) by Tedesco and collaborators. In this case, the evaluation focused on the last published version of the project (ignoring or suppressing the many examples provided of stages in the process)—the last is clearly the most complex and accomplished in the series. The process stages leading up to the last are assumed to be presented as demonstrations of the method, rather than as movements in a finalized output.

Geere and Quick have specialized in making “sonification podcasts” for their series Loudnumbers (https://www.loudnumbers.net/). Each program typically starts with a pedagogic explanation of the design strategy, which effortlessly translates into listening tips (i.e., providing a legend for how the listener can extract meaning from the sounds). In “The End of the Road” (p19), they built on Pape Møller's laboriously collected time series data on insect population density on a rural road in Denmark, and more recently (p10), they turned data from traditional ice measurements in a village in Alaska into techno music.

Crawford and George released projects in 2013 and 2015 that translated the global rise in temperature that in its sonic style leans on classical Western music. Included in the corpus is a cello solo piece (p06). Interestingly, the projects were published in the form of videos where, after an introduction by the authors, the sonification is performed while visualization of the same data appears on screen, as a sort of subtitle or visual support to help the public associate what they hear with what is perhaps a more familiar sensory modality.

The heritage of acoustic orchestral Western music is also the choice of Guda (p11), Twet (p32), and Sawe & Oakes (p29). A similar strategy is also used by Foo in “Too Blue—Mapping Coastal Louisiana's Land Loss with Music” (p09) though with creole-style marching band music.

Working with meteorological records and predictions covering large geographical areas between 2013 and 2016, Lindborg released several instances of “Locust Wrath,” adapting the original multi-channel immersive installation to different contexts, such as dance performance (Lindborg, 2018), sculptural auditory display (Lindborg, 2015), and participatory installation (Lindborg and Liu, 2015). Two are included in the corpus (p15, p16) together with a recent installation piece, “Stairway to Helheim,” (Lindborg, 2022) that fuses abstract and concrete sonic materials with cross-synthesis in a sonification of historical weather records of Hong Kong over 138 years (p17).

Many projects employ long historical time series. In p05, the most Abstract-Informatical in the corpus, Chafe used synthesized sounds to convey the correlation between rising CO_2_ levels and the increase in temperature, using data records from five centuries, from 1666 to 2016. In p25, Quinn represented climate data from the last 110,000 years in a music composition with MIDI instruments.

### 4.3. Aesthetics of data art and scientific communication

Having a unified analysis method for a range of sonic artifacts from the fields of electroacoustic music (e.g., concert and multimedia compositions) and sonification (e.g., software earcons and sys- tem monitoring designs) facilitates interrogation of aesthetic and effectiveness. Vickers underlines that the principles of the former are applicable onto the latter; the primary concern lies with the design of auditory displays and the effectiveness of sonification for the discovery of meaning in data, and more generally for communication. He urges practitioners in the field of sonification to carefully study the principles of electroacoustic music composition, arguing that music and auditory display share important attributes: “it is at these intersections that dialogue and interrogation may take place.” However, he does not equate one with the other, noting that there are “artifacts present in each of music and sonification that are not present in the other… one such is the intellectual content of compositions” (quotes from Vickers and Hogg, 2006, discussed in Lindborg, 2019, p. 44).

Sonification–visualization techniques must not be aestheticized to the point that scientific criteria are neglected. In the context of science communication, researchers have pointed out that “data sonification need not necessarily be musical in nature, and many scientifically-useful auditory graphs are not particularly musical, or even pleasant to listen to. There are some rationales for abstracting the sonification… abstraction can bring some interesting choices to the communicator” (Sawe et al., 2020). In this context, “interestingness” should be understood as a precise psychological concept (Silvia, 2005). As pointed out by Bonet (2021), the term aesthetics does not necessarily denote something “beautiful” or “pleasing,” and sonifications are not necessarily “pleasant,” and that the “aesthetics of a sonification must be linked to its meaning and purpose” (cit. p. 270). Thus, sonification involves several techniques and purposes that, while complying with Kramer's original definition, might also aims to satisfy aesthetic appreciation and to reify the attractiveness of discovery. Aesthetic sonification is not arbitrary. While a scientific approach that emphasizes systematicity and reproducibility is in our opinion fundamental for all data art, successful designs build on ecological perception, i.e., the principle that organisms learn patterns meaningful for survival through exposure and from interacting with the environment (Gaver, 1993; Clarke, 2005; Lindborg, 2018) proposed an “embodied aesthetic framework” to rethink the “relationship between aesthetics and meaning-making in order to tackle the mapping problem” in sonification (Roddy and Furlong, 2014, p.70). The problem they raised becomes apparent in the public's understanding, for example, if they feel that the relationship between data and sound is arbitrary (cf. Vickers and Hogg, 2006). Ultimately, design guidelines are needed to achieve more engaging and effective sonifications.

In this article we have presented a systematic analysis of topics, perceptual characteristics, and aesthetics in a range of sonification and visualization projects. The study aims to contribute to the development of empirically founded design techniques, applicable to climate data communication and other fields. For scientific knowledge to reach people impervious to traditional dissemination methods, researchers in multimodal communication need to explore the relationship between intention strategies, meaning, and aesthetics enabled by extended communication techniques, such as sonification and visualization. This poses challenges for designers of data art aiming to stir audiences into action when faced with the hugely varied and complicated expressions that make up the climate crises. Public engagement with techno-scientific knowledge and its potential societal impact is still an open issue. In this situation, we believe that design informed by research in auditory perception and aesthetics play a central role in creating multisensory experiences that make scientific climate data both meaningful and exciting.

## Data availability statement

The original contributions presented in the study are included in the article/Supplementary material, further inquiries can be directed to the corresponding author.

## Author contributions

PL and SL conceived the study and wrote the Introduction and Discussion Sections. PL conducted the statistical analysis and wrote the Methods and Results Sections. All authors contributed to the analysis, reviewed all parts of the manuscript, and approved the submitted version.

## Supplementary material

Supplementary Data Sheet 1 Spreadsheet with details about the 32 projects, including web links to media and other information.

Supplementary Data Sheet 2 Spreadsheet in “long format” with individually rated scores on 25 rating scales for the 32 projects.

Supplementary Data Sheet 3 Spreadsheet with median ratings on 25 scales of the 32 projects.

Supplementary Data Sheet 4 Spreadsheet containing all post-processed ratings (33 scales × 6 raters × 32 projects), computed variables (7 variables × 6 raters × 32 projects), project descriptors (2 descriptors × 32 projects), and factors.
